# Carers and professionals’ views on using virtual reality in- dementia care: A qualitative study

**DOI:** 10.1177/14713012241272786

**Published:** 2024-08-09

**Authors:** Heema Ajeet Gokani, Andrew Sommerlad, Hiba Jawharieh, Chee Siang Ang, Jonathan Huntley

**Affiliations:** Division of Psychiatry, UCL, UK; Division of Psychiatry, UCL, UK; Camden and Islington NHS Foundation Trust, UK; School of Computing, University of Kent, UK Jonathan Huntley, Division of Psychiatry, UCL, UK; Camden and Islington NHS Foundation Trust, UK and Faculty of Health and Life Sciences, University of Exeter, UK; School of Computing, University of Kent, UK; Division of Psychiatry, UCL, UK; Camden and Islington NHS Foundation Trust, UK and Faculty of Health and Life Sciences, University of Exeter, UK

**Keywords:** dementia, virtual reality, caregivers, healthcare professionals, focus groups

## Abstract

**Background and Objectives:**

Virtual reality (VR) interventions provide immersive, interactive computer-simulated virtual environments. There is interest in their use for people with dementia as they may provide stimulating experiences and improve dementia symptoms and quality of life. However, as more insight is needed about carers’ and clinical professionals’ perspectives to understand how VR may be implemented successfully, we elicited their views on the benefits of, and challenges to, using VR in dementia care.

**Methods:**

We conducted five qualitative focus groups involving 25 healthcare professionals and informal carers with experience of dementia care. Participants received a demonstration of a VR headset and content and were then questioned following a topic guide asking for views on benefits of, and challenges to, using VR for dementia care.

**Findings:**

The main findings addressed the benefits of, and concerns about, the impact and implications of VR on wellbeing, ethics, implementation, caregivers and services. Overall, participants had a positive attitude toward VR and made several suggestions for its future use to enable enjoyable and immersive experiences. Examples included suggestions to personalise VR content to accommodate heterogenous profiles and stages of dementia, co-developing protocols to address health risks and side effects and further investigating shared experiences of VR with caregivers.

**Conclusion:**

Healthcare professionals and informal carers thought that VR had potential to enhance a holistic and personalised approach to dementia care. They suggested changes which could guide future implementation of VR interventions for dementia patients and their caregivers.

## Introduction

Immersive virtual reality (VR) is an emerging technology that allows individuals to interact with computer-simulated digital environments through equipment like VR headsets (Kim et al., 2019). When using immersive VR, participants can engage with full 360° computer-generated 3D content by simply moving their head. This technology generates sensory information, including visual and audio stimuli, creating the illusion of being physically present in the digital environment (Benoit et al., 2015). In dementia care, immersive VR has garnered interest among professionals and researchers due to its potential to offer people with dementia unique sensory and cognitive experiences they could not otherwise access (Rose et al., 2021). Furthermore, it opens up the possibility of shared immersive experiences with carers and family members, facilitating reminiscence and enjoyable interactions (D’Cunha et al., 2019; Huang et al., 2022).

Lawton and Nahemow’s (1973) Environmental Press Model highlights the interplay between an individual’s competency level and environmental demands, emphasising how this interaction influences behaviour and well-being. In the context of virtual reality (VR) interventions for dementia care, this model suggests that designing VR environments to align with individuals’ cognitive and emotional abilities can enhance engagement and mitigate stressors. By crafting VR simulations with familiar and calming settings, tailored interactive challenges, and personalised adjustments over time, these interventions have the potential to dynamically support individuals’ well-being. The model has similarly been applied in the field of dementia by utilising virtual environmental cues to enhance wayfinding for older adults with Alzheimer’s disease (Davis & Ohman, 2016).

In the context of dementia, studies assessing feasibility have generally indicated that VR is safe and well-tolerated (Appel, Appel, et al., 2020; Appel, Ali, et al., 2021; Appel, Kisonas, et al., 2020, 2021; Kim et al., 2019; Kim et al., 2021; Manera et al., 2016; Rose et al., 2021; Strong, 2020). However, they have also highlighted potential health risks and technical barriers (Appel, Kisonas, et al., 2021; Kim et al., 2019; Kim et al., 2021; Rose et al., 2018). Studies have reported adverse health events such as disorientation, motion sickness, and dizziness associated with VR use (Appel, Appel, et al., 2020; Kim et al., 2019; Kim et al., 2021). Technical challenges have revolved around the comfort of VR headsets, particularly for individuals with hearing and vision aids (Appel, Appel, et al., 2020; Appel, Kisonas, et al., 2021). These challenges have been reported to only affect a minority of participants. When examining the effectiveness of VR in in community and institutional memory care settings, a meta-analysis by Kim et al., (2019) revealed small to medium positive effects for VR interventions on physical fitness, cognition, and emotional well-being among individuals with dementia or mild cognitive impairment. Semi-immersive VR interventions demonstrated a greater impact compared to fully immersive ones, and interventions were more effective in community settings than institutions. More recently however, Huang and Yang (2022) discovered no significant impact on the effectiveness of immersive VR reminiscence interventions regarding mood, caregiver burden, and preservation of cognition in elderly patients with dementia. They suggested no long-term effects, only short-term reduction in depressive symptoms.

Furthermore, most studies have primarily focused on individuals with mild to moderate dementia, where cognitive and functional impairment is relatively mild. Two feasibility studies included older adults with advanced dementia, and they found that VR was accepted by individuals with diverse clinical profiles, including those with advanced dementia, limited mobility, and those using hearing or vision aids (Appel, Appel, et al., 2020; Rose et al., 2021). Further research is necessary to address these concerns and barriers related to VR use in dementia care, especially for those with advanced dementia. Previous research has also identified variations in VR preferences based on patient characteristics; for instance, individuals with comorbid dementia and symptoms of apathy tended to derive more enjoyment and benefits from VR activities compared to those with dementia but without symptoms of apathy (Manera et al., 2016). These individual differences and preferences have often been overlooked in VR studies. Furthermore, only one study compared different levels of VR immersion, despite established evidence on the impact of different levels of immersion on the sense of presence and emotional responses (D’Cunha et al., 2019; Lønne et al., 2023). Future research should be guided by expert views on which patient profiles are most likely to engage with and benefit from VR, as well as the optimal delivery methods. Overall, due to methodological complexities, the feasibility and effectiveness of immersive VR in dementia care remain uncertain, necessitating a deeper understanding of VR’s optimal utility in the field.

The VR headset is often paired with a connected laptop or screen, enabling caregivers to assist participants in real-time interaction with the equipment—a feature recommended for incorporation in future studies (Rose et al., 2021). Similarly, Appel, Kisonas, et al. (2021) suggested that VR could benefit family carers and caregivers, as they found enjoyment in observing participants with dementia engaging with VR interventions. Shared VR experiences may bring new benefits to people with dementia and their carers.

Many individuals with advanced dementia reside in care homes and rely on professional caregivers for their daily functioning (Afram et al., 2014). The delivery of VR interventions requires technical expertise and involves potential adverse effects (Appel, Appel, et al., 2020; Appel, Kisonas, et al., 2020, 2021; Kim et al., 2019; García-Betances et al., 2015). Therefore, the implementation of VR interventions relies on caregivers’ willingness and ability to use VR tools. Common barriers and facilitators for staff-led interventions in dementia care typically encompass considerations such as time, resources, personal motivation, and support (Bennett et al., 2021; Saab et al., 2022). This highlights the need for insights from healthcare professionals and informal family carers to inform future VR research and applications in dementia care.

The literature has reported positive receptiveness and willingness among staff to support people with dementia in engaging with VR and participating in the design of VR interventions for dementia care (Appel, Appel, et al., 2020; Coelho et al., 2020; Hodge et al., 2018; Rose et al., 2021; Tabbaa et al., 2019). Considering the pivotal role of professionals and carers in dementia care and the likely support needed for implementing VR in this context, it is essential to gain insights into their perspectives on its utilisation. To our knowledge, a limited number of studies have qualitatively investigated and compared the opinions of health and social care professionals and carers concerning the benefits and challenges of shared experiences of VR in dementia care, especially in regard to improving enjoyment and general wellbeing. Exploration of these perspectives would enable optimisation of VR delivery for future testing of VR in randomised controlled trials (RCTs) for people with dementia and their caregivers.

### Aim and Objectives

The aim of this study was to explore the perspectives of carers and healthcare professionals concerning the use of VR in dementia care. The objectives were:

- To investigate carers’ and professionals’ perspectives on the benefits and risks of using VR technology in dementia care.
- To identify carers’ and professionals’ perceived barriers to and facilitators of the use of VR in dementia care.
- To compare the views of informal family carers and professionals.

## Methods

### Setting and Participants

#### Inclusion Criteria

Participants were recruited for the study based on the following inclusion criteria: aged 18 years and/or above; fluent in English; have experience in dementia care, such as holding the role of a current family carer, care home staff, and/or clinician.

### Recruitment

Participants were recruited using convenience sampling from the following settings:

- A group of care homes based in Kent, UK (Avante Care Homes Kent & Residential Homecare Support). Avante Care Homes is a non-profit organisation that supports over 1,000 older people through registered nursing and dementia care homes, home care, and well-being support services.
- Age UK Camden Dementia Befriending Service in London, UK, which works with volunteer befrienders who visit socially isolated older people living with dementia or memory impairment.
- Clinicians with experience in dementia care at University College London, Barnet, Enfield and Haringey Mental Health National Health Service (NHS) Trust, and Camden and Islington NHS Foundation Trust.

The study was advertised through direct email contact with the above organisations and individuals and using Twitter.

To align with prior research (Guest et al., 2017), we aimed for 3–5 focus groups, each with 4–6 participants (Krueger & Casey, 2000), ensuring data saturation. This approach, involving diverse expertise and experiences across the 3–5 groups, guaranteed comprehensive representation in dementia care (Guest et al., 2017). With 4–6 participants in each group, we fostered in-depth conversations and a secure environment for open opinions. Our goal was 20-25 participants, with ongoing recruitment until reaching theoretical saturation.

### Procedures

#### Demonstration

At the beginning of the focus groups, one researcher (HJ) demonstrated and explained VR. Participants were then invited to use and interact with the META Quest 2 VR headset to familiarise themselves with the device if they wished. All participants were shown 360° videos of landscape (e.g., a beach or cathedral) for two to five minutes.

Previous studies have suggested VR can trigger disorientation, dizziness, and motion sickness, mainly in association with clips of sudden movement and extreme gaming (D’Cunha et al., 2019). We minimised the risk by only showing fixed and neutral clips and had a protocol to safeguard the safety of distressed participants. This enjoyable and non-threatening VR exposure made it consistent with a VR experience which may be offered to people with dementia, allowing participants to give their views on its application in dementia care.

#### Focus Groups

We used a semi-structured interview approach during focus groups and designed a guide that explored participants’

1. Past and current experiences with using VR.
2. Views about benefits and risks of VR in dementia care.
3. Views about facilitators and limitations of VR in dementia care.
4. Ideas and recommendations regarding future applications of VR in dementia care.

We also used open-ended probing to explore participants’ responses in greater depth. The focus groups took place in person in university facilities or office locations in charities or care homes. Participants were reimbursed for travel. One researcher HG conducted focus groups face-to-face, co-facilitated by HJ or JH and audio-recorded them. HG then transcribed all recordings, and anonymised them.

### Data Analysis

Following transcription, HG analysed each focus group of participants using thematic analysis on NVivo 10. A method for analysing qualitative data that entails searching across a data set to identify, analyse, and report repeated patterns (Braun & Clarke, 2006). The research team met regularly to ensure a thorough and compressive process to develop themes that accurately represented the dataset. To facilitate reflexivity, HG, a psychology graduate discussed her reflections regularly with the research team which included two old age psychiatrists (AS, JH), a lecturer in computing (CSA) and a PhD student (HJ), who also reviewed transcripts and discussed and revised codes though, in line with Braun and Clarke guidance (Braun & Clarke; 2022), we did not evaluate inter-rater agreement. We followed Braun and Clarke’s 6-step framework for reflexive thematic analysis for rigorous analysis of the data: Familiarisation, initial coding, searching for themes, reviewing themes, defining and naming the themes and producing the final report (Braun & Clarke; 2006). We compared the views of participants with different roles within dementia care (care home staff vs family carers vs clinicians).

### Ethics

Approval was obtained from the ethics committee of University College London (22575.001).

## Results

Participants’ characteristics are shown in Table 1. We recruited 25 participants across five focus groups. The focus groups lasted from 36 minutes to 57 minutes. Most participants were female (N = 22, 88%). Age ranged from 19 to 81 years (mean = 50.43, SD =16.60). Most participants were White (N = 24, 96%), and almost half the participants had more than ten years of experience caring/working with people with dementia (N = 12, 48%). Full details are summarised in Table 1.

Two focus groups were attended by staff from care homes, two were attended by clinicians and one had family members and volunteer carers. Results are organised into three sections:

1) Views on the benefits of using VR in dementia care.
2) Concerns about using VR in dementia care.
3) Suggestions of future use of VR in dementia care

**Figure 1:**
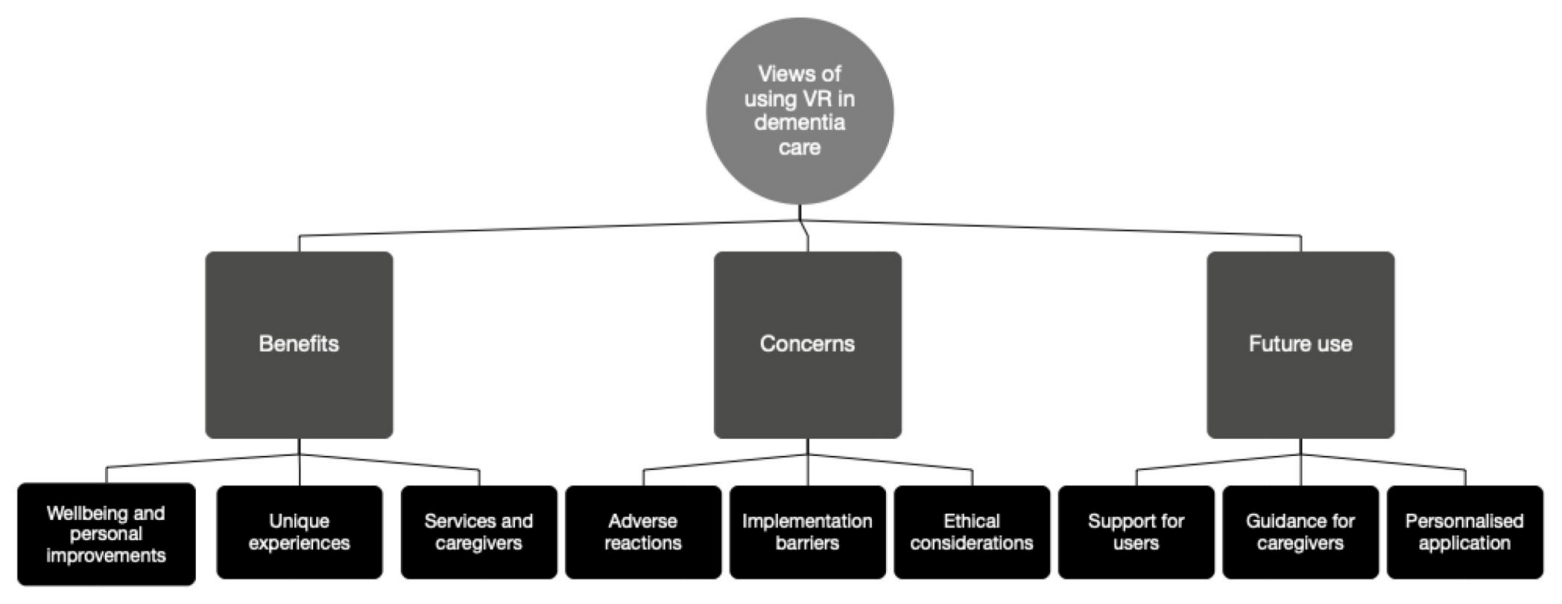
Structure of Themes About the Use of Virtual Reality in Dementia Care

The key themes and findings are discussed below (figure 1).

### Benefits of VR in Dementia Care

#### Wellbeing and Personal Improvements for People with Dementia

Many participants expressed a positive attitude about how VR interventions could contribute to the wellbeing of people with dementia by improving mental health. Participants mentioned how interventions could have an anxiety-reducing effect and be used to support conditions encountered in dementia, such as grief, anxiety, agitation, depression, loneliness, and insomnia.

*‘I think maybe if patients were feeling agitated or having difficulty sleeping or having anxiety then VR might have a place*.*’ (P1, female doctor)*

Participants thought VR interventions could be a non-pharmacological option to stimulate people with dementia and keep them active. They thought the immersive feature of VR could be efficient in keeping people’s attention during therapeutic activities.

‘I think it’s more likely to engage attentional systems.’ (P2, male doctor)

Participants also discussed that VR’s ability to immerse oneself in a different world makes the differences between other forms of entertainment such as going to the cinema, watching online videos, or looking at album photos. One participant viewed VR as an upgrade from cinema.

‘Sometimes watching videos, in care homes, it can be a very busy noisy place, which can be quite distracting […] this is very much immersive, you are very much there.’ (P3, female care home staff)

Many participants said that people with dementia may be more engaged in their tasks and have better outcomes such as staying active and having enhanced cognitive functioning. One speculated that it may potentially slow down cognitive deterioration for those in the early stages.

‘If we started using it quite regularly with people who are on early onset, it could actually slow down the things… so it didn’t evolve so quickly.’ (P4, female care home staff)

Most participants in each group said VR may facilitate social communication and interaction of people with dementia. Care home staff in particular, mentioned that older people living in care homes often felt lonely and had language or cultural barriers impairing communication. Participants suggested that immersive VR experiences exploring new cities or places related to their life could facilitate reactions from people with dementia and help start conversations. Participants added that VR could encourage them dementia to engage in more meaningful social interactions.

‘It can help them communicate … give them something to talk about their experiences with you.’ (P5, female care home staff)

Some respondents explained that VR may provide fulfilment and a purpose in life again for people with dementia. Two participants referred to VR as an immersive experience bringing joy and enjoyment. Other participants also added that VR could be empowering if used to enable people with dementia to share their memories and teach others about their life.

‘They’ll feel much happier and brighter in themselves. They’ll be getting much more fulfilment… enjoying life rather than just existing.’ (P6, female care home staff)

#### Unique Experiences for People with Dementia

Many respondents explained that dementia makes it challenging to live life as before, so they felt optimistic about how VR technology could remove some of the limitations (e.g., restricted mobility). VR would bring new opportunities, like travelling virtually.

‘This is a whole different ballgame to say, well, ‘where are we going to go today?’… It’s a real experience. I think VR is really suited for people whose reality is much more restricted.’ (P2, male doctor)

Participants added that VR would offer opportunities to create unique memories with the family, especially those living at a distance. People with dementia could use VR with their families as a shared experience or receive VR content from them, strengthening family connections. All participants also saw the benefits of using it as a tool for reminiscence exercises so that people with dementia could reconnect with their memories, loved ones, and places.

‘…they would like to see their homes, places and universities so on. And I think it will help people get their youthfulness back.’ (P7, female family carer)

A few participants highlighted that VR could serve as a form of escapism, allowing people with dementia to take a break from their current issues.

‘…they could have that opportunity of escapism.’ (P5, female care home staff)

#### Impact on Services and Caregivers

Some participants suggested that using VR in dementia care can improve services, as it may offer an option that reduces medication use and moves away from an institutionalised style of care. Participants explained that the realistic, immersive and relaxing environment created by VR can offer a more engaging environment for services that tend to be overwhelming or noisy.

‘You can reduce the drug taking, perhaps you could reduce all of that chemical intervention, just by spending time with something like this.’ (P4, female care home staff)

A few participants also discussed that shared experiences of VR would allow carers and professionals to engage more actively with people with dementia. Two professional carers suggested that it would feel satisfying to offer such a unique experience to people with dementia.

‘…it’s not only going to benefit the residents, but you would benefit too. Because you’re going to go home and feel that you made such a difference to somebody’s life.’ (P8, female care home staff)

Finally, participants discussed that VR could also help staff manage challenging behaviour of people with dementia and use it as a tool to distract or refocus them.

‘…some of the behaviours that are difficult to manage where people are distressed… as using it as a kind… of refocusing people with dementia and kind of distracting them from what’s going on.’ (P9, female care home staff)

Family carers added that this would help them reduce some of their burdens and guilt they experience when caring for someone with dementia.

‘…it could alleviate some guilt when you can’t go on holiday and you can’t take your mother, because they’re too fragile.’ (P10, female care home staff)

### Concerns About Using VR in Dementia Care

#### Adverse Reactions to VR

Several participants were concerned about VR causing people with dementia to feel disorientated. Respondents discussed that if the VR exposure was too realistic, patients with dementia might confuse the digital world with reality.

‘…people will actually believe that we are at the beach… So, they will confuse VR with the real experience.’ (P10, female care home staff)

However, one participant suggested that, as people with dementia may already feel confused because of their condition, VR might not reinforce this feeling. There were opposing views about whether confusing VR with reality would encourage people with dementia to physically move or not. Some respondents said it would be an additional risk, as someone could hurt themselves if they started moving with the VR headset. However, others responded that the VR experience demonstrated to them didn’t feel realistic or interactive enough to provoke any physical actions.

‘One resident often thinks that he’s much younger, that he can actually still walk so he tries and get out of his wheelchair. So, my worry is that he would be doing exactly the same thing with the VR.’ (P11, female care home staff)

Further concerns were about how immersive experience and specific visuals could trigger negative feelings, such as fear, sadness, anxiety or feeling of claustrophobia.

‘It could make somebody quite weepy for an instance or it could bring on some agitation or some unwanted behaviour later on.’ (P12, female care home staff)

Many participants debated whether there would be an additional health risk if VR was introduced to more advanced stages of dementia. Some participants said that the feeling of disorientation might be more prominent for people at advanced stages, and one clinician talked about the possibility of experiencing nausea and dizziness. However, others considered VR a low-risk intervention and potentially one of the only ways to stimulate people at more advanced stages.

Some participants concluded that how people with dementia react to VR will be specific to each person, depending on their health and preferences.

‘Maybe it would be suitable for people with advanced dementia… It is not dangerously realistic. So, it should be fine.’ (P13, female clinical psychologist)

#### Implementation Barriers

All groups brought up issues of implementation that are often seen in care homes, which are partly related to staffing limitations and resources. Participants explained that, although a care home might have the resources to deploy VR, staff might stop using it after some time. Other concerns were about considering VR as substitute for interaction when it should be used as a tool supporting meaningful engagement between caregivers and people with dementia. The care home staff explained that it was up to activity coordinators and care staff to lead the VR interventions proactively.

‘Is it a substitute for interaction and temptation for care homes not to do much interactive stuff?’ (P2, male doctor)

Both care home staff and clinicians added that family members should be involved in VR interventions given the benefits VR could bring to their relationships. However, two family carers were concerned VR would be an added burden to them, and facilitation should happen within a care home setting instead.

‘We don’t want to spend so much time… it just seems like another thing to do.’ (P14, female family carer)

#### Ethical Considerations

Most respondents discussed the issues around gaining consent to use a VR headset with people with dementia. Participants discussed that this would involve multidisciplinary meetings, input from family members to make best interest decisions. However, two family carers talked about not feeling comfortable taking a decision on behalf of their partner, and that the preference of their partners might change from day to day. Therefore, given the unpredictability of behaviours that dementia presents, gaining consent could be a challenge.

Clinicians raised ethical concerns about employing technological interventions with patients who are agitated, have diminished cognitive abilities, or sensory impairment.

‘There’s question about I think people’s capacity to understand what’s going to happen to them… So, people who are non-verbal or have limited language capacity, I think is very specific to dementia.’ (P15, female doctor)

Participants mentioned possible concerns around data confidentiality if the VR sessions were to be recorded.

### Future Use of VR

#### Support for Users

Participants said it would be important to provide support for people with dementia using VR and introduce the technology slowly to allow people with dementia to get familiar with it by either having a trial period or letting them watch others use it (peers and staff).

‘I would bring it, I wouldn’t put it on, I would talk about it or leave it until they get interested.’ (P16, male befriender volunteer)

Participants also discussed that people with dementia will require to be supervised by a carer when using VR and receive guidance throughout to minimise the risk of disorientation and adverse reactions.

‘The moment it sits on my head there it was. No warming up, there’s no introduction you’re just there… So, I think what you were saying about the preparation and sitting beside it is really important.’ (P17, female family carer)

A few family carers suggested that VR could be used independently by the people with dementia and said additional support should be provided to do that in the future.

#### Guidance for Caregivers

A few participants suggested carers would need guidance on how to set up and use VR. Some suggested training, bullet pointed guides, or having a helpline to contact in event of technical issues. (e.g., hardware, internet connectivity).

‘…a mix of one-to-one live training sessions and what to expect and … additional support sent to you so that if there were new things being uploaded onto the app…’ (P2, female care home staff)

Some participants suggested training would be needed about supporting people with dementia using VR, including how to read body language, facilitate VR, and manage adverse effects. Two participants suggested a protocol outlining optimal use and potential side effects, as would be the case with a medication.

‘…how to support the person… if the research shows certain things, how you would deal or cope with it.’ (P14, female family carer)

Two family carers and care home staff suggested sessions with team members about past experiences using VR with people with dementia to provide a supportive space for caregivers and help with implementation.

‘I’d like to see some collaboration between all the homes in how it’s benefiting residents.’ (P12, female care home staff)

#### Personalised Application of VR

All participants were favourable towards using VR in dementia care in the future, but emphasised wanting VR interventions personalised to the preferences, personalities, and needs of the people with dementia, which may change during the short- or long-term.

‘I think it needs to be built from personalised care planning. What works for the person, what do they actually enjoy in all these videos.’ (P13, female clinical psychologist)

Participants also discussed the idea of offering VR interventions for entertainment, tailored to individual preferences such as personal, musical, and cultural interests. Some envisioned features like multi-user interactions and virtual VR cafes, online concerts, or personalized videos provided by families. However, they acknowledged potential design and technical challenges in efficiently adapting traditional media formats to VR content at a cost-effective scale.

‘I know there was a gentleman from Croatia, he wants to hear songs from there, so I think it might be very interesting for him.’ (P16, male volunteer carer)

## Discussion

This qualitative study explores the perspectives of clinicians, professional care staff, and informal family carers regarding immersive VR for people with dementia. VR was perceived as feasible and acceptable. Participants generally held positive views, seeing its potential for enjoyment and enhancing wellbeing. Many also believed VR could benefit caregivers and foster social connections through shared experiences. However, concerns arose about VR’s impact on wellbeing, implementation barriers, and ethical implications in dementia care. Furthermore, we noted contrasting opinions among different stakeholders. These discussions helped us identify key considerations for future VR use, including providing support for both individuals with dementia and caregivers, and developing more person-centred VR applications in dementia care.

### Benefits of VR

The current findings are consistent with previous research indicating that VR is perceived as a feasible and acceptable tool for dementia care (Flynn et al., 2003; Moyle et al., 2018). Participants highlighted common benefits observed in the VR literature within dementia care (Flynn et al., 2022). They viewed VR technology as an enhancement over photographs and movies, offering escapism, happiness, fulfilment, and empowerment to individuals with dementia. Our participants emphasised how VR provided unique pleasurable moments that were otherwise inaccessible, underscoring the significance of “in the moment” experiences. While prior studies have acknowledged the limited lasting outcomes on wellbeing and cognition associated with VR use in dementia, they emphasise that this should not diminish the overall value of the experience (Flynn et al., 2022; Walden & Feliciano, 2022). Instead, our study contributes to the literature by emphasising the positive aspects of VR in embracing the present moment and valuing the immediate experiences of individuals living with dementia (Keady et al., 2022).

Furthermore, our participants suggested that VR could help manage behavioural and psychological symptoms and cognitive impairments, which has some support in the literature (Appel, Kisonas, et al., 2020; Kim et al., 2021; Sayma et al., 2020, Keady et al., 2022). Participants suggested that these benefits to physical and mental health could come from the immersive features and sense of presence elicited by VR, which could potentially enable people with dementia to engage more meaningfully in activities than traditional methods, as confirmed by Appel, Ali, et al. (2021). Engagement has been shown to be a protective factor of dementia (Almeida-Meza et al., 2021) and to have positive effects on general wellbeing (Clarke et al., 2020). The immersive feature and its effect on engagement could therefore facilitate the benefits VR brings to wellbeing. Randomised controlled trial evidence is needed to confirm these suggestions.

A unique aspect of VR that we investigated was the use of shared VR experiences for people with dementia and their carers. Overall, participants viewed this opportunity positively, believing VR could enhance communication skills and foster meaningful social interactions. They considered it a unique way to strengthen connections between individuals with dementia and their caregivers. There was even speculation about the potential for virtual attendance at social events like day centres. While no experiments have directly measured the impact of shared VR experiences on the social outcomes of individuals with dementia, prior research has discussed the perceived benefits of VR on social connectedness and the potential for social VR spaces in dementia care (Hodge et al., 2018). Given the positive attitude towards these advanced functions, future developments in VR could explore these possibilities within dementia care.

### Barriers and facilitators

Introducing VR in clinical services would require consideration of the implementation issues known to limit the effectiveness of other non-pharmacological options in dementia care (Gitlin et al., 2021). General challenges of VR include reduced face-to-face communications, education, service structure, cost, users’ attitudes, and safety (Baniasadi et al., 2020), which were frequently mentioned in focus groups. Existing guidance suggests a focus on education and training, inclusion of multidisciplinary teams (clinical, research, financial, lived experience, and IT), and creating gold standard guidelines for the development of VR (Gitlin et al., 2021). Our study emphasises the active role of caregivers, specifically care home staff, in the planning and management of implementing VR interventions and related challenges. Moreover, Moyle et al. (2018) and Flynn et al. (2003) noted that personal preferences and whether VR was facilitated or not could influence the perceived acceptability of the intervention and how far it engaged people with dementia. Some examples of facilitation from our study included, providing topics and flow to guide the user through the VR experience; provide instructions on reading body language to identify risks; and having a manual outlining how to manage side effects When introducing VR to individuals with dementia, participants also suggested demonstrating its use and allowing them to become familiar with it at their own pace, emphasizing the importance of personalised usage of VR. Similarly, Walden and Feliciano (2022) introduced the VR headset by demonstrating its use and allowing participants to wear the headset according to their preference (with or without straps). Facilitating factors should therefore be taken in consideration when addressing the feasibility of VR.

Our participants stressed the importance of providing person-centred and individualised VR experiences. They highlighted the necessity for interventions to address the psychological needs and specific cognitive abilities of individuals with dementia (Hansen, 2017). These suggestions align with Lawton’s Environmental Press model (Lawton & Nahemow, 1973), which underscores the importance of environmental factors in influencing behaviour and well-being. By creating tailored VR environments that complement individuals’ cognitive abilities and reduce environmental stressors, these interventions have the potential to dynamically support individuals’ well-being. The current approach to intervention design has shifted towards a more holistic perspective on living well with a dementia diagnosis, extending beyond a narrow focus on cognitive functioning (Appel, Ali, et al., 2021). People with dementia, actively engaged in recent VR intervention design, have advocated for personalised VR activities, with researchers involving them in the VR development process (Hodge et al., 2018; Kalantari et al., 2022). Furthermore, there is growing recognition of the importance of integrating meaningful and individually targeted activities into dementia care (Hobson, 2019). However, implementing personalised VR solutions can entail significant expenses, including developing content tailored to individual preferences, ensuring seamless integration with dementia care plans, and providing ongoing technical support. While the potential benefits are promising, addressing these financial challenges is essential to make this innovative approach more accessible and widely available to those in need.

### Stakeholders’ opinions

Overall, participants generally had similar views on VR in dementia care, with some differences noted. Clinicians (doctors and psychologists) expressed more ethical concerns and implementation challenges in care home and clinical settings. There were debates about whether VR might cause disorientation and confusion in people with dementia, given their difficulty adjusting to environmental changes (Førsund et al., 2018). Future research could investigate using familiar and pleasant memories in VR to reduce potential distress. Some existing VR-based entertainment interventions reported minor confusion issues (Coelho et al., 2020; Kalantari et al., 2022). It’s crucial to monitor this concern in future research, especially for individuals at higher risk of disorientation, like those with spatial disorientation or delirium.

This debate links to further discussion about ethical concerns on obtaining consent and using VR technology on people at more advanced stages of dementia or with reduced sensory abilities (e.g., mute, visual impairments). One limitation in the field of VR is the lack of representation from people with dementia at more advanced stages and with additional disabilities. Ethical concerns have been acknowledged by professionals in the field (Baniasadi et al., 2020), and current guidance consists of providing training and awareness to caregivers and ensuring a safe development of the technology (Parsons, 2021). Clinicians and staff members in our study suggest that formal and informal carers could take a lead on implementing VR interventions within dementia care and receive various forms of ongoing support to address these concerns.

Both clinicians and professional carers highlighted the importance of VR in promoting social engagement and therefore emphasised the need to involve family members in shared experiences of VR, however family carers discussed the impact on carer burden more than the other groups, and the apprehension of using new technology on their loved ones. However, the sample size of informal caregivers in this study was limited. There is therefore a need to understand caregivers’ perspectives on making technology-related decisions for their loved ones in dementia care.

### Strengths and Limitations

The strength of this study is the diversity of its sample, representing varying roles and levels of expertise within dementia care. All participants received the same demonstrations of VR and explanations of its use in dementia care as a form of entertainment and had the opportunity to try the VR headset; therefore, they had a similar level of understanding of the immersive form of VR.

Several limitations should be noted. Despite their diverse occupational backgrounds, most participants were white females, which could limit generalisability. Inclusion of more ethnically diverse groups could give deeper insight into how VR tools can be adjusted to cultural beliefs and preferences. While our goal was to achieve data saturation, we encountered limited representation from family members, potentially resulting in an incomplete understanding of their perspectives. Combining informal caregivers with formal caregivers and healthcare professionals in our study might have obscured significant differences in their caregiving roles and experiences, influencing their viewpoints. For instance, family caregivers typically provide ongoing, emotionally demanding care which may be more likely to result in stress, burden and anticipatory grief, whereas professional caregivers may offer sporadic support with less emotional investment or long-term dedication. Future research should explore the level of care provided by each group for a more comprehensive analysis. Furthermore, participants had little to no experience of using VR within dementia care, given VR is a novel tool in dementia treatment, recruiting participants with relevant experience was challenging. Our participants’ opinion relied on and was limited to clinical and personal knowledge. However, our VR demonstration and use of focus groups minimised this bias by ensuring exchange of knowledge and expertise among participants. Future research would benefit from interviewing participants who have tried VR with their relatives or patients with dementia.

Qualitative work can be affected by the views and experiences of the interviewers, so we considered the individual opinions of our multidisciplinary research team at every stage of the project and documented personal reflections of values, interest, and insights. Additionally, we acknowledged that participants agreeing to take part in the focus group may have held more favourable views about VR in dementia care, which we considered during reflexivity.

### Reflexivity

HG has a psychology background and a strong interest in digital mental health, and therefore carried pre-existing assumptions into this study, especially related to ethical concerns of VR. Anticipating apprehensions about introducing technology to older individuals due to past interactions, HG added supplementary questions to interviews to clarify these worries. Additionally, our data collection benefited from the involvement of HJ, a Ph.D. student already specialising in VR at one of our recruitment sites. This added depth to our discussions. While one recruited organisation and HJ had pre-existing interest in VR, other participating organisations had no previous experience or stated interest in VR. The team recognised the need to balance between empathetic engagement and maintaining a professional stance, ensuring that emotions and assumptions did not overshadow participants’ narratives. The same applied during data analysis. To manage this, HG engaged in continuous reflective discussions with supervisors and team members to examine emerging data patterns and interpretations of themes, to minimise the influence of personal bias

### Implications and Future Research

The current findings could contribute to the future implementation and development of VR within dementia care and suggest that VR could support a holistic approach to such care by offering different patient-centred experiences. Our study encourages the future implementation and development of VR to consider the input of diverse caregivers to overcome implementation barriers. However, as there is currently no consistent evidence that VR confers any clinical benefit, it cannot be recommended yet as a clinical intervention.

Evidence from RCTs is required to strengthen evidence for VR use and our study suggests ways in which VR could be optimised for future testing. This includes addressing technical challenges and providing comprehensive training for setting up and facilitating VR sessions. To ensure safety and minimise risks, future interventions should incorporate sensory indicators to monitor potential adverse effects such as agitation and dizziness. Moreover, establishing clear protocols for safe entry, exit, and de-escalation is essential to prevent disorientation and derealisation. The costs and benefits of personalising VR content and usage should be revisited given the heterogeneous nature of dementia presentations. Family carers should be involved and there is a need for trials to investigate the impact of shared experiences of VR on personal outcomes such as self-esteem, social engagement, and sense of fulfilment for people with dementia and their carers. As per the participants’ feedback, the realistic and immersive attributes of VR distinguished it from other forms of entertainment or existing care methods for individuals with dementia. These features hold significant potential to provide novel opportunities that were previously restricted for individuals with dementia. In light of these insights, future VR interventions may prioritise the recreation of immersive and meaningful experiences for this population, with the aim of enhancing their engagement and enjoying the present moment. This will inform the future provision of care targeted towards the psychological needs of people affected by dementia and improve quality of life.

## Supplementary Material

Appendix

## Figures and Tables

**Table 1:** Demographic characteristics of participants (n=25)

	Mean	SD
Age (n = 21)	50.4	16.6
	N	%
**Gender**		
Female	22	88.0
Male	3	12.0
**Ethnicity**		
Asian or Asian British	1	4.0
White	24	96.0
**Number of years’ experience caring/working** **with/for people with dementia**		
Less than 5 years	9	36.0
5 to 10 years	3	12.0
More than 10 years	12	48.0
Missing	1	4.0
**Main role**		
Family member/relative caregiver	3	12.0
Care home healthcare assistant	4	16.0
Social worker	3	12.0
Doctor	4	16.0
Other healthcare professional	2	8.0
Care home activity coordinator	4	16.0
Other care home staff	5	20.0

## Data Availability

The datasets generated during and/or analysed during the current study are available from the corresponding author on reasonable request.
