# Supplementary material for: Carers and professionals’ views on using virtual reality in- dementia care: A qualitative study
Source: Dementia (London). Author manuscript; Available in PMC 2025 Aug 15. (PMC7618012; doi:10.1177/14713012241272786)
Supplement: Appendix [file EMS207648-supplement-Appendix.pdf]

Appendix

Table of criteria for reporting qualitative research

Questions	Answers	Location in manuscript
**Domain 1: Research team and reflexivity**		
**Personal Characteristics**		
1.Interviewer/facilitator<br>Which author/s conducted the interview or focus group?	HA, HJ, JH	
2. Credentials<br>What were the researcher's credentials? E.g., PhD, MD	MSc, PhD, MBBs	
3. Occupation<br>What was their occupation at the time of the study?	Student, PhD student, Principal Research Fellow	
4. Gender<br>Was the researcher male or female?	Female, Female, Male	
5. Experience and training<br>What experience or training did the researcher have?	HA has received qualitative research methods training by the MSc. She has worked on two previous qualitative studies and has experience interviewing participants on sensitive health-related topics.<br>HJ is studying at a doctoral level and has previous experience conducting qualitative study and deploying VR technology in care homes.<br>JH is a consultant psychiatrist and research fellow with extensive experience in and expertise in the field of dementia. JH has also conducted various qualitative studies.	
**Relationship with participants**		
6. Relationship established<br>Was a relationship established prior to study commencement?	Yes. JH and AS invited their colleagues to attend the focus group. HJ is a PhD student funded by Avante Care Home, which was one of our recruitment sites.	p20-21

7. Participant knowledge of the interviewer<br>What did the participants know about the researcher? e.g., personal goals, reasons for doing the research	Participants were briefed on the purpose of the study and understood that it was a research project for HA's MSc. Ethical approval from UCL had been granted, participants reviewed the participant information documentation prior to giving their written informed consent to participate.	
8. Interviewer characteristics<br>What characteristics were reported about the interviewer/facilitator? e.g., Bias, assumptions, reasons, and interests in the research topic	HA has an interest in developing digital technology to support long-term neurological conditions in safe and ethical ways. HA was interested in knowing people's views on the limitations related to the ethics of using VR. HA had assumptions that VR would not be received well within the context of dementia care and older adults before the start of the study. HJ is completing a Ph.D about developing and deploying VR interventions in care homes for older adults.	p20-21
**Domain 2: study design**		
**Theoretical framework**		
9. Methodological orientation and Theory<br>What methodological orientation was stated to underpin the study? e.g., grounded theory, discourse analysis, ethnography, phenomenology, content analysis	Thematic analysis	p6
**Participant selection**		
10. Sampling<br>How were participants selected?	Participants were recruited using convenience sampling methods and were selected based on the inclusion criteria of the study.	p5
11. Method of approach<br>How were participants approached? e.g., face-to-face, telephone, mail, email	Participants were approached through emails and social media. Some participants were recruited at Avante Care & Support headquarters during their VR training	p5
12. Sample size<br>How many participants were in the study?	There were 25 participants	p7

13. non-participation<br>How many people refused to participate or dropped out? Reasons?	1 person refused to participate during recruitment at Avante Care Home & Support recruitment	
**Setting**		
14. Setting of data collection<br>Where was the data collected? e.g., home, clinic, workplace	UCL facilities, care home and/or charity headquarters	p5-6
15. Presence of non-participants<br>Was anyone else present besides the participants and researchers?	No	
16. Description of sample<br>What are the important characteristics of the sample? E.g., demographic data, date	The majority of participants were female (N = 22, 88.0%). Age ranged from 19 to 81 (mean = 50.43, SD =16.60). Most participants were from a white ethnicity (N = 24, 96.0%) and half of the participants had more than 10 years of experience caring/working with people with dementia (N = 12, 50%). Participants held various roles within dementia care, more details are outlined in Table 1 in the manuscript.<br><br>All focus groups were held between May and July 2022	p7
**Data collection**		
17. Interview guide<br>Were questions, prompts, guides provided by the authors? Was it pilot tested?	Interviews were semi-structured using a topic guide; follow up	p6
18. Repeat interviews<br>Were repeat interviews carried out? If yes, how many?	No	
19. Audio/visual recording<br>Did the researcher use audio or visual recording to collect the data?	Yes, audio recording. The researcher used an encrypted audio recorder and UCL's zoom recording and cloud server services.	p5-6

20. Field notes<br>Were field notes made during and/or after the interview or focus group?	No additional field notes were made.	
21. Duration<br>What was the duration of the interviews or focus groups?	The semi-structured interview durations ranged from 36 minutes to 57 minutes.	p7
22. Data saturation<br>Was data saturation discussed?	No	
23. Transcripts returned<br>Were transcripts returned to participants for comment and/or correction?	No	
**Domain 3: analysis and findings**		
**Data analysis**		
24. Number of data coders<br>How many data coders coded the data?	There was only one coder.	
25. Description of the coding tree . Did authors provide a description of the coding tree?	Yes	
26. Derivation of themes<br>Were themes identified in advance or derived from the data?	Themes were derived from the data.	p6-7
27. Software<br>What software, if applicable, was used to manage the data?	NVivo 10	p6
**Reporting**		

29. Quotations presented<br>Were participant quotations presented to illustrate the themes/findings? Was each quotation identified? e.g., participant number	Yes, participants quotations were presented, and they were identified with a participant number	P8-15
30. Data and findings consistent<br>Was there consistency between the data presented and the findings?	Yes	
31. Clarity of major themes<br>Were major themes clearly presented in the findings?	Yes	P8-15
32. Clarity of minor themes<br>Is there a description of diverse cases or discussion of minor themes?	No	
